# Phylogenomic Analysis of *Cytochrome P450* Gene Superfamily and Their Association with Flavonoids Biosynthesis in Peanut (*Arachis hypogaea* L.)

**DOI:** 10.3390/genes14101944

**Published:** 2023-10-15

**Authors:** Kun Zhang, Yongmei Qin, Wei Sun, Hourui Shi, Shuzhen Zhao, Liangqiong He, Changsheng Li, Jin Zhao, Jiaowen Pan, Guanghao Wang, Zhuqiang Han, Chuanzhi Zhao, Xiangli Yang

**Affiliations:** 1 College of Agricultural Science and Technology, Shandong Agriculture and Engineering University, Jinan 250100, China; zhangkun3___3@163.com (K.Z.); qymwxy@sohu.com (Y.Q.); zhin064@163.com (J.Z.); 2 Institute of Crop Germplasm Resources (Institute of Biotechnology), Shandong Academy of Agricultural Sciences, Shandong Provincial Key Laboratory of Crop Genetic Improvement, Ecology and Physiology, Jinan 250100, China; zhaoshuzhen51@126.com (S.Z.); shzyyxgs@126.com (C.L.); jwpan01@126.com (J.P.); wgh90325@126.com (G.W.); chuanzhiz@126.com (C.Z.); 3 Linyi Academy of Agricultural Sciences, Linyi 276003, China; lysw2005@163.com; 4 Shandong Seed Management Station, Jinan 250100, China; houruis2018@163.com; 5 Cash Crop Research Institute, Guangxi Academy of Agricultural Sciences, Nanning 530007, China; heliangqiong@163.com (L.H.); hanzhuqiang@163.com (Z.H.)

**Keywords:** *A. hypogaea*, cytochrome P450, expression pattern, flavonoid biosynthesis pathway

## Abstract

Cytochrome P450s (CYPs) constitute extensive enzyme superfamilies in the plants, playing pivotal roles in a multitude of biosynthetic and detoxification pathways essential for growth and development, such as the flavonoid biosynthesis pathway. However, CYPs have not yet been systematically studied in the cultivated peanuts (*Arachis hypogaea* L.), a globally significant cash crop. This study addresses this knowledge deficit through a comprehensive genome-wide analysis, leading to the identification of 589 *AhCYP* genes in peanuts. Through phylogenetic analysis, all AhCYPs were systematically classified into 9 clans, 43 gene families. The variability in the number of gene family members suggests specialization in biological functions. Intriguingly, both tandem duplication and fragment duplication events have emerged as pivotal drivers in the evolutionary expansion of the *AhCYP* superfamily. Ka/Ks analysis underscored the substantial influence of strong purifying selection on the evolution of *AhCYPs*. Furthermore, we selected 21 genes encoding 8 enzymes associated with the flavonoid pathway. The results of quantitative real-time PCR (qRT-PCR) experiments unveiled stage-specific expression patterns during the development of peanut testa, with discernible variations between pink and red testa. Importantly, we identified a direct correlation between gene expression levels and the accumulation of metabolites. These findings offer valuable insights into elucidating the comprehensive functions of *AhCYPs* and the underlying mechanisms governing the divergent accumulation of flavonoids in testa of different colors.

## 1. Introduction

Cytochrome P450s (CYPs) represent a superfamily of enzymes that are ubiquitously found across eukaryotes and prokaryotes, spanning animals, plants, fungi, protists, archaea, bacteria, and even certain viruses. This widespread distribution hints at their ancient evolutionary origins [1,2]. Originally, these enzymes earned the name “cytochrome P450” due to their propensity to bind to CO in a reduced state, displaying an absorption peak at 450 nm, and regarded as pigments with heme-containing protein characteristics [3,4]. Subsequent research unveiled their true nature as enzymes facilitating oxidative modifications of diverse substrates, employing oxygen and NAD(P)H as essential cofactors [5]. Today, they are also recognized as monooxygenases, capable of introducing oxygen atoms into hydrophobic molecules, rendering them more active or hydrophilic [6]. In plants, CYPs constitute one of the most extensive enzyme protein superfamilies. Structurally, all known plant CYPs are membrane-bound enzymes, predominantly anchored in the endoplasmic reticulum membrane via a hydrophobic signal sequence at their N-terminus [7]. Presently, following the established CYP450 nomenclature, the plant genome encompasses a total of 10 clans, 74 families, comprising 127 distinct subfamilies [1].

Due to their diverse activities, which encompass decarboxylation, sulfoxidation, hydroxylation, reduction, dealkylation, dehalogenation, deamination, epoxidation, and ring extension, CYPs play indispensable roles in driving plant growth, development, and resilience through various biosynthetic and detoxification pathways [8,9,10]. A multitude of CYPs, serving as structural genes, participate extensively in the metabolism of primary and secondary metabolites, such as flavonoids, phenylpropanoids, coumarins, phenolic esters, terpenoids, alkaloids, lipids, cyanogenic glucosides, glucosinolates, benzoxazinones, isoprenoids, as well as phytohormones and signaling molecules [1,11,12,13]. This vast diversity of families and members within the CYP superfamily underscores their wide-ranging involvement in these biological processes. In the context of our focus on flavonoid biosynthesis pathways, all CYPs involved are confirmed to belong to the CYP71 clan [14].

Flavonoids represent a prominent class of polyphenolic compounds found widely in plants, with over 9000 species identified to date [15,16]. These compounds typically possess a C6-C3-C6 carbon framework, exhibiting structures akin to chromane or chromene. Flavonoids encompass various subtypes, including flavanones, flavones, flavonols, flavanols, isoflavones, anthocyanidins, and proanthocyanidins, with chalcones, dihydrochalcones, and aurones, although structurally distinct, often classified under the broader term of flavonoids [17,18,19]. Flavonoid biosynthesis originates from the breakdown of phenylalanine, undergoing multiple enzymatic conversions to yield chalcone, typically considered the starting point of flavonoid biosynthesis [20,21]. Subsequently, intermediates like chalcone and naringin follow distinct pathways to produce various types of flavonoids [22]. Across these branching pathways, multiple CYPs play crucial catalytic roles. Due to their unique structural features, flavonoids are significant contributors to plant growth, development, and resistance to both biotic and abiotic stresses [23]. Moreover, these bioactive compounds hold increasing value in promoting human health, with numerous flavonoids demonstrating antioxidant, anti-aging, anti-tumor, anti-inflammatory properties, as well as efficacy in treating cardiovascular and cerebrovascular diseases [24,25,26,27].

Cultivated peanut (*Arachis hypogaea* L.) holds a position of paramount importance worldwide, serving as a vital source of both oil and protein for human consumption. Notably, the peanut’s seed coat, often referred to as the testa, boasts a rich nutritional profile, particularly in terms of flavonoids. Colored peanut is an important direction in peanut breeding, and the differentiated biosynthesis and accumulation of flavonoids is the main reason for the color difference in peanut testa. Despite their pivotal role in regulating flavonoid biosynthesis, the comprehensive analysis and genomic identification of the *Cytochrome P450* superfamily genes (*AhCYPs*) in peanut had, until now, remained unexplored. In this study, we embark on a systematic endeavor to identify the members of the AhCYP superfamily, delving into the intricate web of their phylogenetic relationships, chromosomal distribution, gene structures, promoter attributes, and evolutionary connections within the gene family. Moreover, our qRT-PCR findings provide valuable insights into the potential roles of specific *AhCYPs* in steering flavonoid biosynthesis and accumulation during the development of peanut testa. These discoveries not only serve as a foundational platform for a more profound comprehension of the roles and evolutionary trajectories of *AhCYP* genes but also pave the way for further exploration into the molecular mechanisms and regulating testa color traits of AhCYPs in the nuanced synthesis and accumulation of flavonoids in peanuts.

## 2. Materials and Methods

### 2.1. Identification of AhCYP Superfamily Genes

We procured the genome sequences and associated data for *A. hypogaea* from Peanutbase (https://data.legumeinfo.org/data/v2/Arachis/hypogaea/, accessed on 15 April 2023) [28]. The CYP protein sequences of *Arabidopsis thaliana* were obtained from TAIR database (https://www.arabidopsis.org/index.jsp, accessed on 15 April 2023) [29]. Hidden Markov Model (HMM) files for the Cytochrome P450 domain (PF00067) were sourced from the Pfam database (http://pfam.xfam.org/, accessed on 18 April 2023) [30]. Using HMMER3.0 software (http://hmmer.org/download.html, accessed on 18 April 2023) [31] with a query threshold of E-value ≤ 1 × 10^−5^, we identified AhCYP proteins. Potential members were subsequently validated using the NCBI-CDD database (https://www.ncbi.nlm.nih.gov/cdd/, accessed on 21 April 2023) [32] and SMART database (http://smart.embl-heidelberg.de/, accessed on 21 April 2023) [33] to eliminate sequences with incomplete structural domains or redundancy. We calculated the number of amino acids, molecular weight, and theoretical isoelectric point (pI) for AhCYP protein sequences using ExPASy (https://web.expasy.org/protparam, accessed on 25 April 2023) [34].

### 2.2. Phylogenetic Analysis of AhCYPs

Alignment of all AhCYPs and AtCYP protein sequences was conducted using ClustalX with default parameters [35]. The phylogenetic tree was generated via FastTree v2.1.11 (Maximum Likelihood, default parameters). The final tree was visualized and refined using the EvolView v3 tool (http://www.evolgenius.info, accessed on 25 May 2023) [36].

### 2.3. Structural Characterization of AhCYP Family Members

The gene structure of each *AhCYP* was depicted based on the genome sequence and its annotation file using the “Gene Structure View” tool within Tbtools v1.127 [37]. We predicted the conserved motifs of the AhCYPs using MEME v5.5.4 (https://meme-suite.org/meme/doc/meme.html, accessed on 12 May 2023), specifying a maximum of ten motifs and an optimized motif width ranging from 6 to 50 [38]. To analyze *cis*-acting elements within the *AhCYPs*, we employed PlantCARE (http://bioinformatics.psb.ugent.be/webtools/plantcare/html/, accessed on 13 May 2023) [39]. Visualization of the aforementioned data was achieved using Tbtools v1.127.

### 2.4. Chromosomal Location and Collinear Analyses for AhCYPs

We extracted the physical coordinates of *AhCYPs* from the genome annotation file and mapped their locations on the chromosomes using TBtools v1.127. The Multiple Collinearity Scan toolkit (MCScanX) was utilized to investigate gene duplication events and their collinear relationships within the peanut genome and with other species (*Glycine max*, *Arachis duranensis*, and *Arachis ipaensis*). Genome files for *G. max* were downloaded from Soybase (https://www.soybase.org/dlpages/, accessed on 7 May 2023) [40], while those for *A. duranensis* and *A. ipaensis* were obtained from Peanutbase (https://data.legumeinfo.org/data/v2/Arachis/duranensis/, accessed on 7 May 2023; https://data.legumeinfo.org/data/v2/Arachis/ipaensis/, accessed on 7 May 2023) [28]. Additionally, nonsynonymous (ka) and synonymous (ks) substitutions for identified gene pairs were estimated using TBtools v1.127.

### 2.5. Plant Materials

For this study, we utilized two distinct peanut varieties: Yuanza 9102 (Y9102) with pink testa and ZhongHua 12 (ZH12) with red testa. These seeds were sown in the experimental field in 2022 and grown under standard management practices at the Jiyang Experimental Station of Shandong Academy of Agricultural Sciences (SAAS), located in Shandong, China (coordinates: 36°58′34.53″ N, 116°59′1.29″ E).

Testa samples were collected at four different developmental stages from both Y9102 and ZH12, specifically at 15 days after pegging (DAP 15, referred to as S1), 30 days after pegging (DAP 30, S2), 45 days after pegging (DAP 45, S3), and 60 days after pegging (DAP 60, S4). Three biological replicates were performed and each replicate comprised the testa from 10 seeds harvested from at least three plants of the same variety. Subsequently, these samples were rapidly frozen in liquid nitrogen and stored at −80 °C.

### 2.6. Anthocyanin Quantification

To determine anthocyanin content, we employed a modified method based on a previously established protocol [41]. The absorbance of the supernatant was measured at 530 nm and 657 nm using a spectrophotometer (U-3000, HITACHI, Hitachi City, Japan). The relative anthocyanin content was calculated using the formula: 1 unit = (A_530_ − 0.25 × A_657_)/Fresh Weight (g).

### 2.7. Transcription Profiling Based on RNA-Seq Data

In this investigation, we examined the expression of *AhCYPs* using transcriptome data obtained from four materials: ZH12, Y9102, Bulk-red, and Bulk-pink. The latter two comprised mixtures of red testa and pink testa, respectively, in the F4 generation of Y9102 and ZH12 hybridization. These data are accessible via the NCBI website’s Sequence Read Archive (SRA) (https://www.ncbi.nlm.nih.gov/sra, PRJNA886491, accessed on 2 July 2023) [42]. We selected *AhCYPs* with non-zero FPKM (fragments per kilobase of transcript per million fragments mapped) values from the four materials for analysis. The FPKM values underwent preprocessing with log_10_ transformation and were used to generate heatmaps using the R program v4.1.2. Differentially expressed genes (DEGs) were statistically analyzed using Excel, employing the criteria |log_2_FC| ≥ 1 and *p* < 0.05.

### 2.8. Expression Analysis of Desired Gene

Total RNA was extracted from the collected samples using the FastPure Plant Total RNA Isolation Kit (Vazyme, NanJing, China). RNA purity and quantity were assessed through gel electrophoresis and BioPhotometer D30 (Eppendorf, Hamburg, Germany). First-strand cDNA synthesis was carried out using the *Evo M-MLV* RT Mix Kit with gDNA Clean for qPCR (Accurate Biology, Changsha, China). Primer design for each target gene was accomplished using Primer Premier 5 software (Table S1). *AhActin* served as the internal reference gene for data normalization [43]. qRT-PCR experiments were performed using the SYBR Green Premix Pro Taq HS qPCR Kit (Accurate Biology) and an ABI7500 Real Time System (Applied Biosystems, Foster City, CA, USA). Each reaction was conducted in a total volume of 20 μL under the following conditions: 94 °C for 10 min; 94 °C for 15 s, 60 °C for 10 s, and 72 °C for 25 s, repeated for 40 cycles. Three biological replicates were conducted for each sample. Relative gene expression was determined using the2^−∆∆Ct^ method [44].

### 2.9. Statistical Analysis

Student’s *t*-test was performed using Graphpad Prism 8.0 software. A *p*-value cutoff of 0.05 (or 0.01) was used to determine whether the test results were significantly (or extremely significantly) different. The statistical power was calculated using Calculators (www.powerandsamplesize.com/Calculators/, accessed on 8 October 2023), which we consider to be greater than 80%, and the results were reliable. The error bars represented the standard deviation (SD) from independent biological replicates.

## 3. Results

### 3.1. Identifying CYPs in Peanut

**Figure 1:**
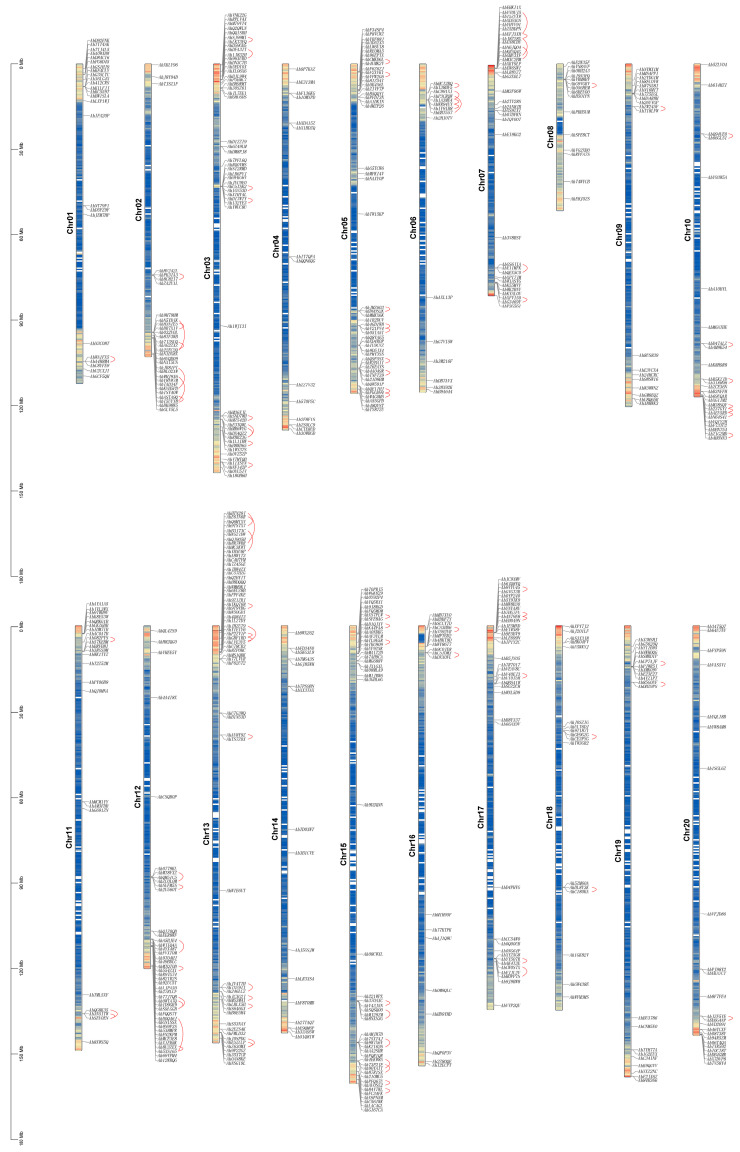
Distribution map of 589 *AhCYPs* on 20 peanut chromosomes. Chromosome lengths (in Mb) are indicated on the left scale bar. Chromosome numbers are displayed on the left side of each chromosome, while gene IDs are shown on the right. Tandemly duplicated genes are highlighted by red lines.

Understanding the *AhCYP* gene family and analyzing their physical and chemical properties forms the fundamental basis for exploring their involvement in various biochemical mechanisms and cellular processes. We initiated this endeavor by conducting a blast search against the *A. hypogaea* genome database, accessible through Peanutbase. Subsequently, after rigorous validation, we successfully identified a total of 589 *AhCYP* genes. These *AhCYPs* were extensively mapped to chromosomes ranging from Chr01 to Chr20. Notably, Chromosome 13 harbored the highest number of *AhCYPs*, totaling 29, closely trailed by chromosomes 03, 15, and 05, each accommodating 24, 22, and 22 *AhCYPs*, respectively. In contrast, Chromosome 02 exhibited a more modest presence, hosting a mere 14 genes (Figure 1, Table S2).

Statistical analysis revealed that the peanut A subgenome (Chr01 to Chr10) comprised a total of 279 *AhCYPs*, while the B subgenome (Chr11 to Chr20) contained 308 *AhCYPs*. Interestingly, the distribution of *AhCYPs* on these two subgenomes exhibited a remarkable symmetry, aligning seamlessly with the well-established high homology between the two subgenomes (Figure 1, Table S3).

Further analysis unveiled notable variations among AhCYP proteins, encompassing differences in length, size, and various physicochemical properties. Amino acid lengths ranged from a compact 79 (as observed in AhC57HZG) to a substantial 1270 (exemplified by AhETWN8F). Molecular weights (MWs) spanned from a lean 9077.20 Da (AhC57HZG) to a considerable 146019.69 Da (AhETWN8F). Theoretical isoelectric points (pI) exhibited diversity, oscillating between a mildly acidic 4.81 (AhPY06B9) and a moderately alkaline 10.29 (Ah6EUZ8B). Meanwhile, protein instability indices (II) fluctuated across the spectrum, ranging from 25.99 (AhPY06B9) to 66.27 (AhWHJ9X6), and aliphatic indices displayed variability from 69.33 (AhVNK22G) to 126.56 (AhQ8VAE5). In terms of the Grand Average of Hydrophobicity (GRAVY), with the exception of 25 members like AhQ8VAE5 and AhLAC4GL, which displayed hydrophobic characteristics, the remainder exhibited properties indicative of hydrophilic proteins (Table S4). These diverse structural features underscore the multifunctional roles played by CYP proteins in peanut growth and development.

### 3.2. Phylogenetic Tree Analysis and Family Classification of AhCYPs

**Figure 2:**
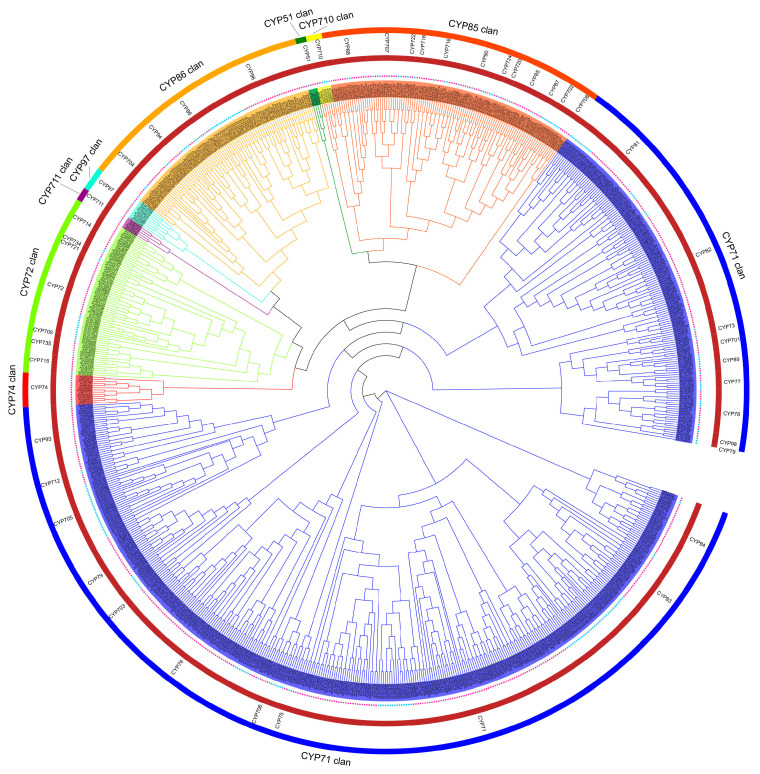
Phylogenetic tree illustrating CYPs from peanut and *Arabidopsis*. Utilizing a maximum likelihood approach, a tree clustered with 589 AhCYPs (marked by red stars) and 235 AtCYPs (marked by blue stars) into 9 clans, 47 gene family (clan names are marked in the outer circle, and gene family names are marked in the inner circle). Each clan denoted by distinct colors. Note: The phylogenetic tree was generated with FastTree v2.1.11 program (Maximum Likelihood) and visualized by EvolView v3 tool.

To gain a deeper understanding of the phylogenetic relationships among the *CYP* superfamily genes, a comprehensive phylogenetic tree was constructed based on the multiple sequence alignment of the 589 AhCYPs and 235 AtCYPs of *A. thaliana* (Figure 2). According to the universally recognized classification standards for CYPs, we systematically categorized the total of 824 CYPs into 9 clans, 47 distinct gene families. In line with other species studied, the peanut and *Arabidopsis* species belonging to A Type, clan71, have the highest number of families and members, with 376 and 146 members in 19 families. It is worth noting that the remaining clan types fall into the non-A category, where CYP51 clan, CYP710 clan, CYP711 clan, CYP74 clan, and CYP97 clan each constitute a single-family clan. In contrast, CYP72 clan, CYP85 clan, and CYP86 clan are designated as multi-family clans. Remarkably, both species encompass all nine clans characteristic of dicotyledonous plants (Table 1).

Notably, while both peanut and Arabidopsis CYPs exhibit a systematic and even distribution within each clan, these two species markedly diverge in the distribution of gene family members. Compared to *Arabidopsis*, CYP702, CYP705, CYP709, and CYP721 family members are missing in peanuts, most notably CYP705, which has 25 members in *Arabidopsis* but 0 in peanuts. In addition, the number of members is unevenly distributed in many gene families, such as CYP93, which has 27 members in peanuts and only one member in *Arabidopsis* (Table 1). These intriguing patterns of gene family expansion and contraction form the genetic underpinning for the divergent synthesis and accumulation of related metabolites.

### 3.3. Analysis of Motifs and Promoter cis-Elements

**Figure 3:**
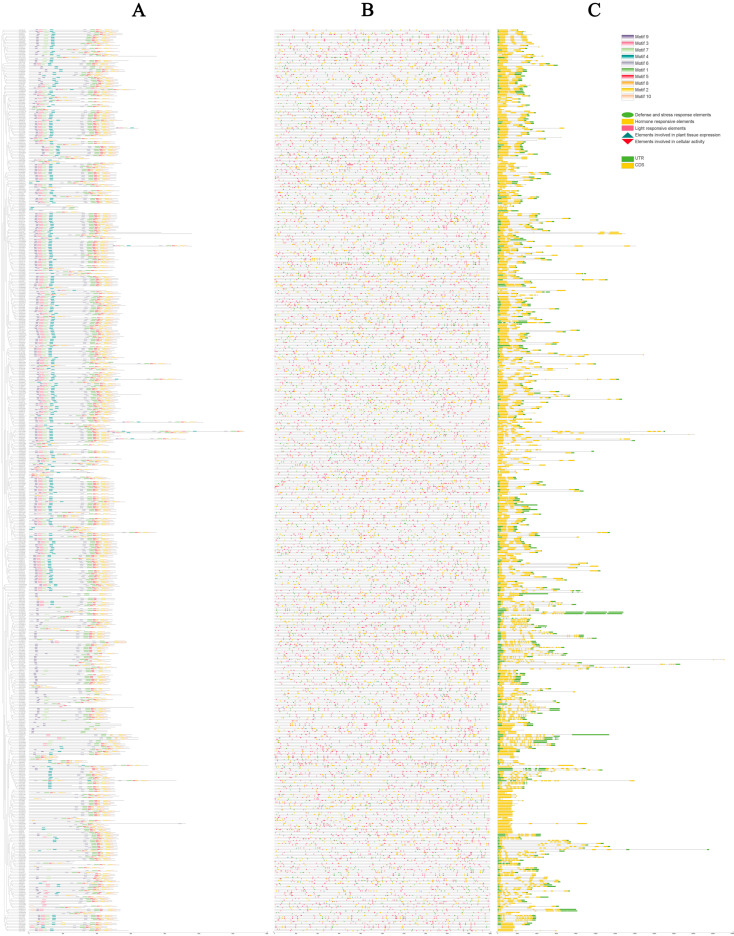
Conserved protein motifs, promoter *cis*-elements, and gene structures of *AhCYPs*. (**A**) Conserved protein motifs. Various colored blocks depict the arrangement of conserved motifs. (**B**) Predicted *cis*-elements within *AhCYPs* promoters. Different shapes and colors represent the different *cis*-element types. (**C**) Gene structure of *AhCYP* genes. CDS, UTR, and introns were represented by yellow boxes, green boxes, and black lines, respectively. Protein length estimation is facilitated by the scale at the bottom.

For a more comprehensive understanding of the commonalities and variations in the gene structures of *AhCYPs*, we conducted a meticulous examination of the conserved domains within AhCYP proteins, leveraging the phylogenetic tree as our guide. The MEME v5.5.4 was employed to unearth conserved motifs within the 589 AhCYP proteins, revealing a treasure trove of insights (Figure 3A, Table S5). These motifs, thoughtfully numbered from 1 to 10, elegantly corresponded to sequences represented in logos 1 to 10 (Figure S1). In consonance with the structural attributes of the CYPs’ conserved domain, we ascertained that the heme-binding signature resided within Motif 2 (N-QDFEFJPFGAGRRICPGISLA-C, 88.62%), the K-helix within Motif 1 (N-ESDJNKLPYLKAVIKETLRLHPPVPLLLP-C, 90.32%), the I-helix within Motif 6 (N-GTDTTAVTJEWAMAELJKN-C, 76.23%), and PERF was meticulously nestled within Motif 8 (N-EEFKPERFLESDIDF-C, 75.21%). Furthermore, it is fascinating to note that the remaining motifs exhibited varying degrees of conservation, with members sharing the same motif combination likely to be associated with similar biological functions.

Promoter *cis*-elements wield a pivotal role in initiating gene expression. In our meticulous exploration, we unveiled a grand total of 66 distinct types of *cis*-regulatory elements residing within the promoter regions of *AhCYP* genes. These elements were thoughtfully categorized into five broad classes, each with its unique role: defense and stress response elements, elements involved in cellular activity, elements involved in plant tissue expression, hormone responsive elements, and light responsive elements (Figure 3B, Table S6). Among these, light-responsive elements emerged as the most abundant and diverse, boasting elements such as TCT-motif, Box 4, and MRE, which collectively accounted for 48.38% of the total. Hormone-responsive elements, encompassing those sensitive to auxin (AuxRE, TGA-element), abscisic acid (ABRE), MeJA (CGTCA-motif, TGACG-motif), ethylene (ERE), gibberellin (GARE-motif, P-box), and salicylic acid (TCA-element), constituted 30.50% of the total. Notably, numerous promoter regions of AhCYP members harbored ARE, DRE core, GC motif, LTR, TC rich repeats, MBS, and WUN motif elements, indicating their pivotal roles in triggering plant responses to conditions such as anoxia, low temperature, drought, and mechanical stress. Furthermore, certain promoter regions exhibited a limited array of cis-regulatory elements related to cellular activity and plant tissue expression, including MSA-like, MBSI, CAT-box, and GCN4-motif. This remarkable diversity of elements suggests that the expression of *AhCYP* family members is intricately regulated by a multitude of factors and adapts to various environmental conditions.

### 3.4. Gene Structures of AhCYPs

In order to understand the characteristics of *AhCYPs*, we analyzed their gene structures, including the intron–exon structure. Our meticulous analysis of gene structures revealed a diverse spectrum of exon numbers, spanning from 1 to 18. The highest number of exons was observed in *Ah570P5C* and *AhF8T0MB*, with 37 other members, such as *AhXKLY96* and *AhWHJ9X6*, intriguingly devoid of introns. Notably, the prevailing structural pattern among *AhCYPs* featured a solitary intron, with a total of 251 members adopting this configuration, constituting 42.61% of the total (Figure 3C). The details are listed in Table S7. The similarity of this pattern was determined by their genetic origin and evolution from the same ancestor.

### 3.5. Duplication, Syntenic, and Evolutionary Analyses of AhCYP Genes

Based on established research, the presence of a chromosomal segment spanning 200 kb or more, housing two or more highly homologous genes, is recognized as indicative of a tandem duplication event [45,46]. In this extensive study, we have meticulously identified a total of 93 tandem duplication events, involving 168 AhCYP genes. These events were distributed across all chromosomes, except for Chr04 and Chr14 (Table S8). Notably, the most frequent occurrence of tandem duplication events was observed on Chr13, Chr15, Chr03, and Chr05, aligning with a symmetrical distribution pattern on both the A and B subgenomes. The interconnected tandemly duplicated gene pairs have been distinctly highlighted by the discerning use of red arcs in Figure 1.

**Figure 4:**
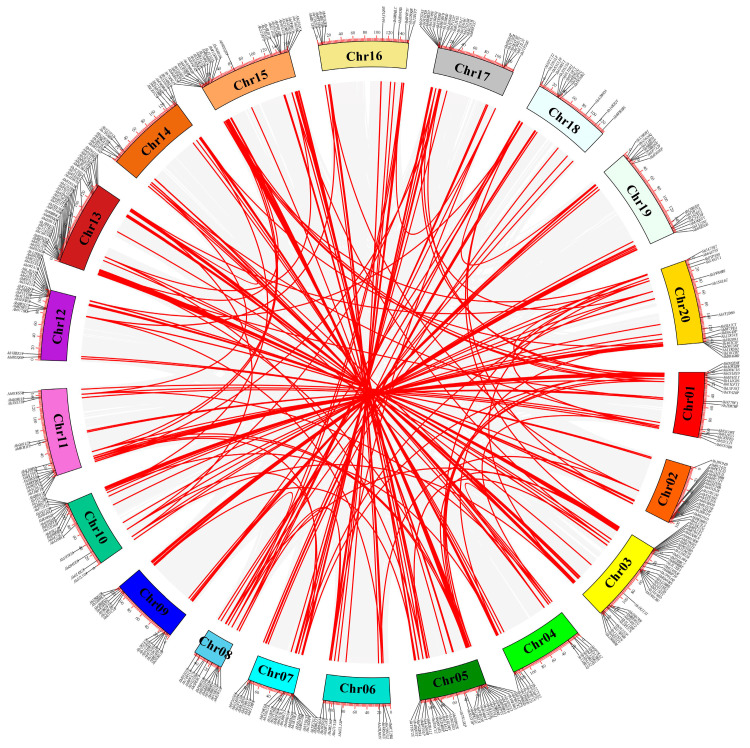
Circos plot illustrating collinearity among *AhCYP* homologous genes. Collinear blocks across the genome are depicted in gray, while duplicated *AhCYP* gene pairs are linked with red curves. Note: This diagram was created using MCScanX in TBtools v1.127.

Segmental duplications lead to the proliferation of duplicated chromosomal blocks within genomes, often coinciding with polyploidization events and chromosomal rearrangements [35]. Across the peanut genome, a total of 245 segmental duplication events, involving 373 *AhCYP* genes, were unveiled (Table S9). These homologous *AhCYP* genes are visually depicted and connected by red curves in the collinear Circos plot presented in Figure 4. In essence, it is the collective interplay of both tandem and segmental duplication events that propels the expansion of the *AhCYP* gene superfamily.

**Figure 5:**
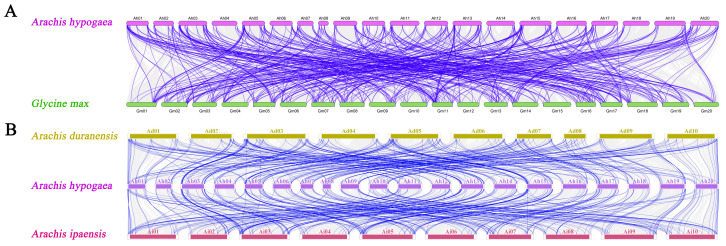
Syntenic relationships of *AhCYPs* across peanut and three representative plant species. (**A**) Comparison between *G. max* and *A. hypogaea*. (**B**) Comparison among, *A. duranensis*, *A. ipaensis* and *A. hypogaea*. Gray lines indicate collinear blocks within *A. hypogaea* and other plant genomes, while blue lines emphasize syntenic *AhCYP* gene pairs. Note: This graph was drawn using MCScanX in TBtools v1.127.

In our quest for a deeper understanding of the evolutionary underpinnings of CYPs within *A. hypogaea* and other related species, we judiciously leveraged a Leguminous model plant (*G. max*) and two *Arachis* species (*A. duranensis* and *A. ipaensis*), widely acknowledged as the wild ancestors of cultivated peanut, for synteny analyses. These findings were subsequently integrated into the comparative syntenic schematics illustrated in Figure 5. In totality, 381 AhCYPs exhibited synteny with *G. max* (209 genes), *A. duranensis* (186 genes), and *A. ipaensis* (178 genes) (Table S10). The shared orthologous gene pairs spanning different species provide valuable insights for conducting pertinent evolutionary investigations concerning *AhCYP* genes.

**Figure 6:**
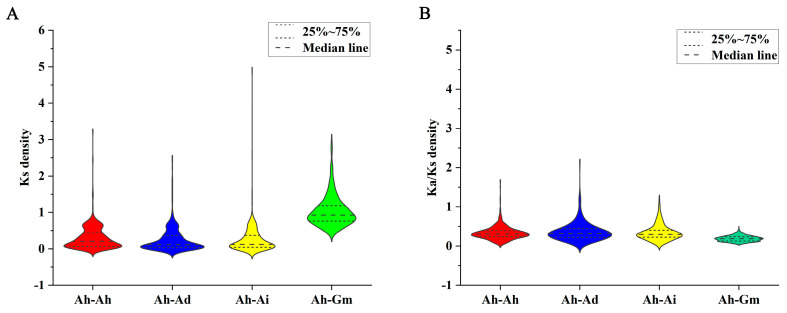
Violin plot representing Ks and Ka/Ks ratios in orthologous *CYP* gene pairs. (**A**) Ks. (**B**) Ka/Ks. Prefixes “Ah”, “Ad”, “Ai”, and “Gm” denote *A. hypogaea*, *A. duranensis*, *A. ipaensis*, and *G. max*, respectively.

In our quest to shed light on the evolutionary constraints that have sculpted the CYPs superfamily, we embarked on analysis of Ka (non-synonymous substitution), Ks (synonymous substitution), and Ka/Ks ratios for the *CYP* orthologous gene pairs across peanut and the three species (Table S11). Notably, our scrutiny of Ks value distributions revealed an alignment of the 25%, 75%, and median lines for Ah−Ad and Ah−Ai, closely mirroring Ah−Ah. However, they stood distinctly apart from Ah−Gm (Figure 6A). Furthermore, it is noteworthy that a significant proportion of orthologous *CYP* gene pairs exhibited Ka/Ks ratios below 1, an indication that the CYP family in peanuts has been subjected to pronounced purifying selective pressure throughout its evolutionary journey (Figure 6B).

### 3.6. Expression Patterns of AhCYP Genes in Peanut Testa

**Figure 7:**
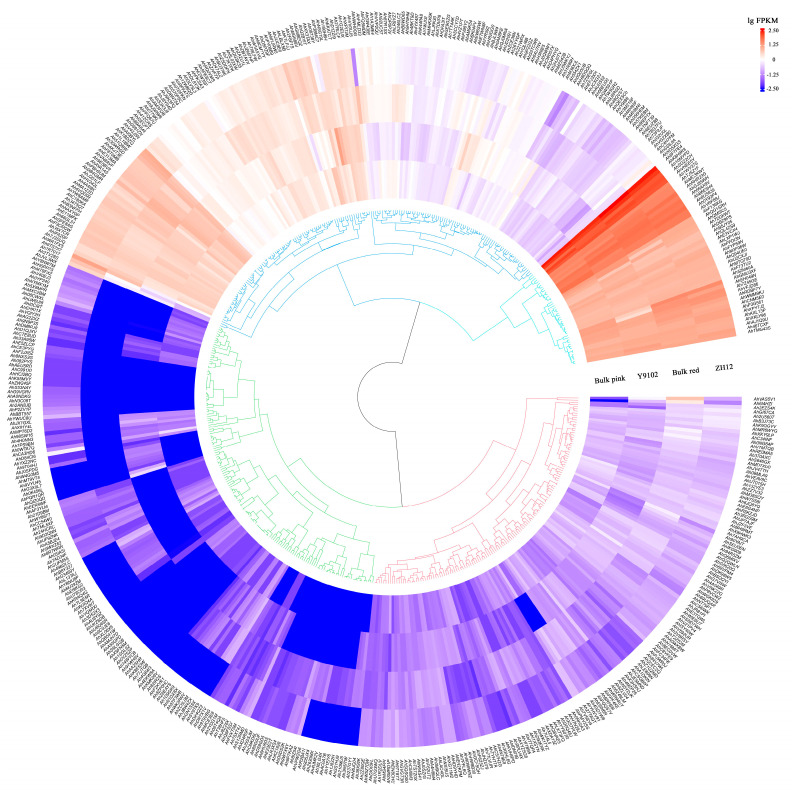
The expression profiles of *AhCYP* genes. Gene expression level is expressed in lg(FPKM). Note: This graph was drawn using the R program v4.1.2.

The gene expression of *AhCYPs* in the testa of Y9102, ZH12, Bulk pink and Bulk red were investigated using the transcriptome data. Available expression data are presented in Table S12. Our study found that 517 *AhCYPs* were detected to be expressed in four materials, exhibiting specific expression patterns. These gene expression differences are significant, and these differences are presented on the heatmap with different shades of color blocks (Figure 7). 

In direct comparison with Y9102, ZH12 exhibited the upregulation of 134 *AhCYPs* and the downregulation of 89 *AhCYPs*. Similarly, when compared with Bulk pink, 124 *AhCYPs* were found to be upregulated, and 93 were downregulated in Bulk red. A finding emerged when juxtaposing Y9102 and Bulk pink: a total of 42 *AhCYPs* were substantially upregulated, while 20 *AhCYPs* experienced significant downregulation in both ZH12 and Bulk red (Table S12). These discernible differences in gene expression may be intricately linked to the disparities in testa color observed between these two distinct varieties.

### 3.7. Expression of AhCYPs Involved in Flavonoid Pathway during Peanut Testa Development

**Figure 8:**
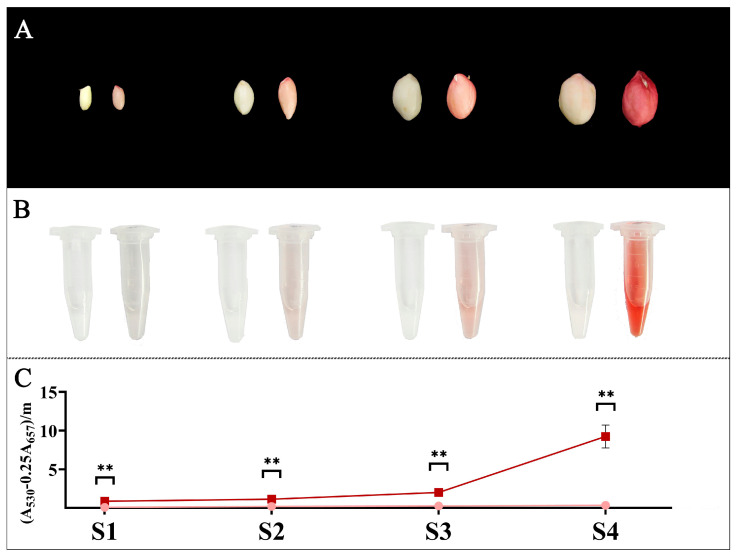
Phenotypes and anthocyanidin content of testa in four stages (S1–S4) of Y9102 and ZH12. (**A**) The phenotypes of Y9102 and ZH12 testa at different stages, with Y9102 on the left and ZH12 on the right in a pair of seeds. (**B**) The visual display of anthocyanins extracted from the testa of Y9102 and ZH12, Y9102 on the left centrifuge tube and ZH12 on the right. (**C**) Determination of anthocyanin content. The dark red line is ZH12, and the pink line is Y9102. Asterisks indicate significant differences in anthocyanidin accumulation between ZH12 and Y9102 in this period (** *p* < 0.01; *t*-test).

Transcriptome data from only one developmental stage of the testa cannot accurately reflect the role of relevant *AhCYPs* during development. Therefore, we divided the development of peanut testa into four stages, namely S1–S4. According to the phenotype and anthocyanin assay results, we found that the anthocyanin content in red testa was significantly higher than that in pink testa during the four stages of peanut testa development, and from 10.51 times in S1 to 27.12 times in S4, the difference in anthocyanin accumulation gradually increased (Figure 8, Table S13). However, there was no significant difference in total flavonoid content between pink and red testa in S4 [47], suggesting that different flavonoid substances accumulated in different colored testa.

According to previous research results, combined with phylogenetic and transcriptome analysis studies, we identified eight enzymes involved in flavonoid biosynthesis pathway in peanut CYPs, involving a total of 21 genes. Their detailed information is shown in Table 2. Notably, cinnamate 4-hydroxylase (C4H) is a key enzyme in phenylalanine metabolism upstream of the flavonoid pathway, and flavonoid 3′-hydroxylase (F3′H) is considered to be a key structural gene through anthocyanin synthesis, but in fact, it is also involved in the synthesis of flavones and flavonols. 2-hydroxyisoflavanone synthase (IFS), isoflavone 2′-hydroxylase (I2′H), isoflavone 3′-hydroxylase (I3′H), flavonoid 6-hydroxylase (F6H) and 3,9-dihydroxypterocarpan 6a-hydroxylase (D6aH) belong to isoflavone branching pathway. In the flavone branching pathway, flavone synthase II (FNS II) plays a pivotal role.

In order to explore the function of *AhCYP* genes in the mechanism of flavonoid differentiated accumulation, pink and red testa materials of four developmental stages (S1–S4) were used for determining the expression level of candidate genes by qRT-PCR. The quality of the extracted total RNA was higher (Figure S2), and the melting curves of each of the 21 candidate genes showed a single peak (Figure S3), which ensured the accuracy of the qRT-PCR results.

**Figure 9:**
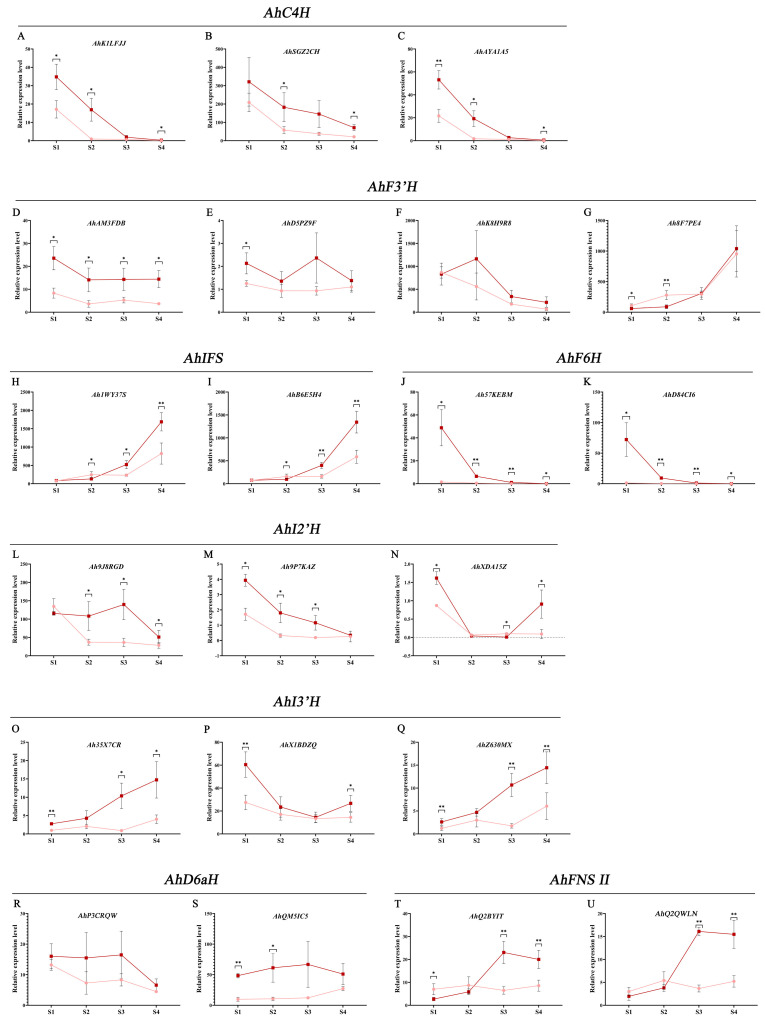
qRT-PCR analysis of *AhCYP* genes in different development periods of testa. (**A**–**C**) *cinnamate 4-hydroxylase* (*C4H*) genes in peanut; (**D**–**G**) *flavonoid 3′-hydroxylase* (*F3′H*); (**H**,**I**) *2-hydroxyisoflavanone synthase* (*IFS*); (**J**,**K**) *flavonoid 6-hydroxylase* (*F6H*); (**L**–**N**) *isoflavone 2′-hydroxylase* (*I2′H*); (**O**–**Q**) *isoflavone 3′-hydroxylase* (*I3′H*); (**R**,**S**) *3,9-dihydroxypterocarpan 6a-hydroxylase* (*D6aH*); (**T**,**U**) *flavone synthase II* (*FNS II*). The dark red line represents changes of gene expression in ZH12 testa, while the pink line represents changes in Y9102. Stage 1 (S1): 15 days after pegging (DAP 15); S2: DAP 30; S3: DAP 45, and S4: DAP 60. Asterisks indicate significant differences in relative expression level between ZH12 and Y9102 in this period (* *p* < 0.05, ** *p* < 0.01; *t*-test). Relative expression level is compared to *Ah35X7CR* in S1 period, set as 1.00.

*AhC4H* has three homologous genes, namely *AhK1LFJJ*, *AhSGZ2CH*, and *AhAYA1A5* (Figure 9A–C). According to the relative expression, with the continuous development of testa, these three genes showed similar expression patterns, and the expression levels decreased rapidly. *AhK1LFJJ* and *AhAYA1A5* decreased to almost no expression during S3 and S4 periods, while *AhSGZ2CH* still maintained certain expression. In addition, the expression of three *AhC4H* genes in red testa was higher than that in pink testa. At the same time, the expression level of *AhSGZ2CH* was several times to ten times that of *AhK1LFJJ* and *AhAYA1A5*, which should be the *AhC4H* that plays a major role in testa.

Four F3′Hs were found in peanut, which are believed to be involved in multiple branching pathways such as anthocyanins, flavones, and flavonols, namely *AhAM3FDB*, *AhD5PZ9F*, *AhK8H9R8*, and *Ah8F7PE4* (Figure 9D–G). According to the expression level, *AhK8H9R8* and *Ah8F7PE4* should be the main genes regulating the synthesis of flavonoids in testa. Interestingly, the overall trend of *AhK8H9R8* in red and pink testa from S1 to S4 is decreasing, while *Ah8F7PE4* is sharply increasing.

There are many AhCYPs in isoflavone synthesis pathway. The first structural gene of the isoflavone pathway, *IFS*, has two genes in peanut, *Ah1WY37S* and *AhB6E5H4* (Figure 9H,I). The expression of these two genes is high, and both are major genes. The expression patterns of the two genes were similar, from S1 to S4, and the expression levels were significantly up-regulated in both types of skin. In addition, the two genes in S3 and S4 were significantly/extremely significantly higher in red testa than in pink testa. The expression levels of two *AhF6H* genes, *Ah57KEBM* and *AhD84CI6*, were significantly higher in the red testa during the S1 period than in the pink testa (Figure 9J,K). However, they both sharply decreased during the S1 to S4, and were very low in both materials during the S4 period. Among the three *AhI2′H* genes, the expression level of *Ah9J8RGD* was tens of times that of *Ah9P7KAZ* and *AhXDA15Z*, and it should be the dominant gene (Figure 9L–N). *Ah9J8RGD* was significantly down-regulated in the early stage of pink testa, while it was down-regulated in the late stage of red testa. *Ah9J8RGD* in red testa was significantly higher than that in pink testa during S2–S4. AhI3′H has three genes, namely *Ah35X7CR*, *AhX1BDZQ*, and *AhZ630MX* (Figure 9O–Q). The expression patterns of *Ah35X7CR* and *AhZ630MX* were similar. During the S1–S4 period, the expression levels of these two genes were up-regulated in both pink and red materials, and the expression levels in red materials were significantly/extremely significantly higher than those in pink. The expression of *AhX1BDZQ* tended to be down-regulated. Similarly, the expression of *AHX1BDZQ* in red materials was higher than that in pink. The expression levels of the two *AhD6aH* genes, *AhP3CRQW* and *AhQM5IC5*, were not significantly related to the developmental stage of the testa, but the expression levels of the two genes in the red testa were higher than those in the pink testa during the same period (Figure 9R,S).

The first enzyme in the flavone synthesis pathway, FNS II, has two genes encoding this enzyme in peanuts, namely *AhQ2BYIT* and *AhQ2QWLN* (Figure 9T,U). The expression patterns of these two genes are the same, and in the red material, they are significantly up-regulated in the S1–S3 period, while slightly down-regulated in the S4 period; there was no significant change in the pink material, maintaining low levels of expression. During the S3 and S4 stages, both genes were significantly higher in the red material than in the pink material. The relative expression level of above genes is listed in Table S14.

## 4. Discussion

### 4.1. The Evolution of AhCYP Superfamily: Diversity and Expansion

The CYP gene superfamily, one of the largest in plants, plays a pivotal role in catalyzing a diverse range of reactions involved in growth, development, and secondary metabolite biosynthetic pathways. Systematic identification and study of the *CYP* gene superfamily are paramount. It is reported that there were 246 *CYPs* in *A. thaliana*, 356 *CYPs* in rice (*Oryza sativa*) [48], 236 *CYPs* in grape (*Vitis vinifera*) [49], 285 *CYPs* in Tartary buckwheat (*Fagopyrum tataricum* (L.) *Gaertn.*) [50], and 263 *CYPs* in maize (*Zea mays*) [51]. In our study, we conducted a comprehensive genome-wide analysis of *CYP* gene superfamily members in cultivated peanuts, identifying a total of 589 *AhCYP* genes (Table S2). The size of the *CYP* gene superfamily is influenced by polyploid evolutionary processes. For instance, allohexaploid wheat has as many as 1285 *CYPs* [51]. In our study, allotetraploid peanuts also exhibited a relatively large number of *CYPs*. Interestingly, genome size does not appear to dictate *CYP* quantity. Cultivated peanuts, with a genome size of 2.4 Gb, have 589 *AhCYPs*, whereas *A. thaliana*, with a genome size of only 125 Mb, possesses 246 *AtCYPs*. This diversity among members encompasses variations in length, size, physicochemical properties, and other attributes, underscoring the functional diversity of this gene superfamily.

To standardize nomenclature and classification of CYPs, the P450 nomenclature committee (dnelson@uthsc.edu) established a universal system based on protein sequence identity and phylogeny [51]. Plant CYPs have been categorized into gene families spanning CYP71-CYP99 and CYP701-CYP999 based on their sequence identities [52] From a broader phylogenetic perspective, plant CYP families and subfamilies are consolidated into ten independent clans based on ancestral lineage [53]. Each clan is named after the smallest family within it, encompassing the 71 Clan, 72 Clan, 85 Clan, 86 Clan, 51 Clan, 74 Clan, 97 Clan, 710 Clan, 711 Clan, and 727 Clan, with the latter being unique to monocotyledonous plants [48,54]. In our study, we constructed phylogenetic trees for cultivated peanuts and *A. thaliana*, categorizing clans and families according to these established rules. While peanut and *Arabidopsis* share similar clans and families, subfamilies and member counts differ significantly (Figure 2, Table 1). These distinctions contribute to the selective regulation of metabolic pathways. For instance, isoflavone biosynthesis is a unique pathway in legumes. Subfamilies CYP93A, CYP93C, and CYP81E, closely associated with isoflavone biosynthesis, feature numerous members in peanut (Table 2), soybean [55] and alfalfa [56]. In contrast, these subfamilies are absent in *Arabidopsis*, and there are no reports of isoflavone synthesis in this plant. Therefore, like other plants, the peanut CYP superfamily has evolved for specialization, resulting in differential synthesis and accumulation of metabolites, aligning with its species origin, ecological adaptation, and survival strategies [57].

CYPs share highly conserved motifs, including the heme-binding signature (FXXGXRXCXG), the K-helix (EXXR), the I-helix (AGXD/ET), and the PERF motif, all of which are vital for their catalytic activity [58]. Conservative motif analysis verified that most AhCYPs contain the conserved domains of CYP, including the heme-binding signature, K-helix, I-helix, and PERF motif (Figure 3A, Table S5). Notably, specific motifs unique to particular groups of *AhCYPs* were discovered alongside these conserved motifs, implying specialized biological functions within specific *AhCYP* groups. Promoter *cis*-elements play pivotal roles in gene expression regulation. Genes with distinct *cis*-regulatory elements in their promoter sequences may exhibit different expression patterns. Our study detected a total of 66 types of *cis*-regulatory elements in *AhCYP* gene promoter regions, categorized into five groups. This high diversity in *cis*-regulatory elements suggests functional divergence at the transcriptional level (Figure 3B, Table S6). The intron–exon gene structure is a hallmark of gene superfamily evolution. Plant *CYPs* are believed to have polyphyletic origins, with A-type P450s (Clan 71) typically featuring one highly conserved intron, while non A-type branches exhibit varying intron numbers [59]. In our study, we observed certain patterns in the intron–exon gene structure of *AhCYPs*, with 251 *AhCYPs* containing only one intron, of which 218 belong to Clan 71, accounting for 86.85%. Some gene families have certain similarities in intron–exon gene structure, such as CYP710 members without intron, while *CYP701* members all have seven introns (Figure 3C, Table S7). Intron–exon structure appears to be a useful tool in establishing the evolutionary relatedness of CYPs, which may help in predictions of their function [60]. 

Gene duplication emerges as a pivotal force driving plant evolution. Among the mechanisms facilitating gene duplication, tandem and segmental duplication events take center stage [61]. Tandem duplication events was mainly for rapid adaptation to external pressure, and segmental duplication events was directly related to the origin of polyploidy in plants [62]. The results showed that there are 93 tandem duplication gene pairs and 245 segmental duplication gene pairs in the *AhCYP* superfamily, both of which showed strong expansion characteristics (Table S8, Table S9). It is worth highlighting that different plant species exhibit varying patterns of gene duplication. For instance, grapes predominantly undergo tandem duplication, whereas segmental duplication takes precedence in rice [49,63]. In estimating speciation timeframes, the distribution of Ks values among homologous gene pairs from distinct species is frequently employed, with the Ka/Ks ratio serving as a barometer for assessing selective pressures [64,65,66,67]. Our findings lend support to the notion that *A. hypogaea* originated from natural distant hybridization between *A. duranensis* and *A. ipaensis* [68]. Furthermore, the divergence between *G. max* and *Arachis* occurred significantly earlier (Figure 6A). The Ka/Ks values for each *AhCYP* homologous gene pair underscore a robust purifying selection process throughout evolution (Figure 6B).

### 4.2. Expression Pattern Analysis of AhCYPs Participated in Flavonoid Biosynthesis

An important biological function of CYPs is to play a crucial role in the flavonoid biosynthesis [57]. Our attention was drawn to three *AhCYPs*, namely *Ah8F7PE4*, *AhK8H9R8*, and *AhSGZ2CH*, with the highest FPKM values among all four materials in the transcriptome dataset. These genes are structural components of the flavonoid biosynthesis pathway, encoding one *AhC4H* and two *AhF3′H* enzymes, respectively (Table S12 and Table 2). This also indicates that during the development stage of peanut testa, the biosynthesis of various flavonoids is very active. In our investigation, we identified a total of 21 genes encompassing eight enzymes within the CYPs associated with the flavonoid pathway (Table 2). These enzymes play pivotal roles in the anthocyanin, flavonoid, flavonol, and isoflavone pathways. It is noteworthy that, in general, these enzyme-encoding genes are not unique; typically, there are one or two predominant genes. Whether this is due to tissue specificity remains unclear. Additionally, it is evident that the expression of these genes was generally higher in the red testa compared to the pink testa (Figure 9).

Moreover, we found that all genes have stage specificity and their expression changes adhere to distinct regulations. Isoenzyme genes frequently exhibit similar expression patterns, especially among the major genes. For instance, *AhK1LFJJ*, *AhSGZ2CH*, and *AhAYA1A5*, all of which are *AhC4H*, displayed their highest expression levels during the S1 period in both pink and red testa, followed by a rapid and consistent decline in subsequent stages. Notably, their expression levels in red materials consistently exceeded those in pink materials (Figure 9A–C). Similarly, the expression levels of *AhIFS*, *Ah1WY37Sc* and *AhB6E5H4* witnessed a significant upregulation from S1 to S4 (Figure 9H,I). In contrast, certain genes exhibited differential expression patterns in pink and red testa. For instance, the two *AhF6H* genes displayed peak expression in the S1 stage in red material, followed by a sharp decrease, whereas they were nearly inactive from S1 to S4 in pink material (Figure 9J,K). As for the two *AhFNS II* genes, they initially increased in red testa, reaching their zenith at the S3 stage and subsequently experiencing a slight decline. Conversely, no significant changes were observed in pink testa (Figure 9T,U).

Of particular interest is the expression of the two major isozyme genes, *AhK8H9R8* and *Ah8F7PE4*, within the *AhF3′H*. *AhK8H9R8* maintained a consistently declining trend, while *Ah8F7PE4* exhibited the opposite pattern, displaying a significant upregulation from the S1 to S4 stages in both red and pink materials (Figure 9F,G). F3′H modifies the B-ring of the flavonoid backbone at the C3′ position [69]. In the flavonoid biosynthesis pathway, F3’H catalyzes various biochemical reactions. In the anthocyanin branching pathway, it catalyzes dihydrokaempferol to dihydroquercetin; in the flavonol branching pathway, it catalyzes kaempferol to quercetin; and in the flavone branching pathway, it catalyzes Apigenin to Luteolin [70,71]. Therefore, our speculation hinges on the functional specialization of these two genes, enabling their participation in distinct branching pathways, but there is currently no direct evidence to prove it.

Additionally, our results hint at an intriguing pattern: the early expression of upstream genes such as *AhC4H* and the late expression of downstream genes such as *AhIFS* and *AhFNS II*. This pattern aligns with the metabolite synthesis process. Nevertheless, it is imperative to acknowledge that this observation, while intriguing, lacks definitive evidence and warrants more comprehensive validation.

### 4.3. Expression of AhCYPs and Differential Accumulation of Flavonoid Metabolites

Peanut testa is rich in various flavonoids. Scientists posit that peanut testa, rich in these bioactive substances, holds considerable potential for enhancing human health and is consequently being increasingly explored for the development of functional foods or the extraction of purified pharmaceutical compounds [72,73,74]. Research has unveiled that the content of proanthocyanidins in the peanut testa can reach remarkable levels, constituting up to 17% (*w*/*w*) of its dry weight, a significantly higher proportion than that found in grape seeds (5–6.5%). Proanthocyanidins are pivotal compounds in grape renowned for their contributions to the antioxidant properties and anti-aging effects of wines [75]. Another set of flavonoids that has garnered considerable attention in peanut testa is anthocyanidins, responsible for imparting distinct colors to peanut seeds [76]. Compared with ordinary pink peanuts, consumers perceive red peanuts as having higher nutritional value, leading to a premium market price. Red peanut is also an important direction for high-quality peanut breeding. It was found that the red testa trait was determined by the content of anthocyanins [76]. 

In recent years, with the continuous deepening of research, researchers have found that not only anthocyanins, but also significant differences in the types and content of other flavonoids contained in different colored testa. For example, compared with other colored peanuts, in addition to containing a large amount of anthocyanins and proanthocyanidins, a variety of flavonols, rutin in particular, are also specifically accumulated in the testa of red peanuts at an impressive concentration of 0.20 mg/g (fresh material) [47]. Consequently, red peanut testa holds promise as a future source for extracting natural, bioactive rutin, recognized for its antiviral, antioxidant, and anti-inflammatory properties [77,78,79].

**Figure 10:**
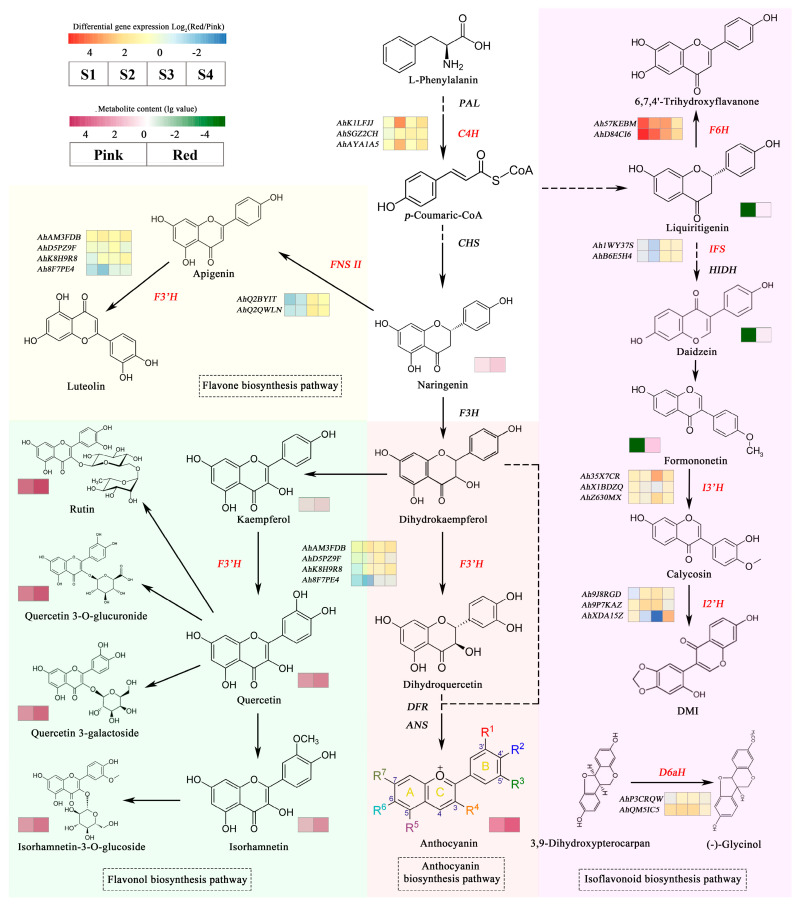
Schematic of flavonoid biosynthesis pathway in peanut. The *AhCYP* genes involved in the pathway are highlighted with red names, with expression heatmaps next to the names. The expression difference is obtained by taking log_2_ as the ratio of ZH12 to Y9102 in this period. The metabolite content is log_10_ value, which is indicated by different shades of color blocks next to the metabolite. Different flavonoid branching pathways are represented by different background colors.

In this study, we amalgamated previous research findings on flavonoid metabolomics in pink and red peanut testa [47] with the expression levels of selected *AhCYPs* for a comprehensive analysis, as depicted in Figure 10. Significantly higher levels of liquiritigenin, formononetin, and daidzein, all isoflavones, were found in red testa compared to pink, in line with the elevated expression of *AhCYPs* genes *AhIFS*, *AhI2′H*, *AhI3′H*, *AhF6H*, and *AhD6aH* within the isoflavone biosynthesis pathway in red testa when contrasted with pink. Moreover, flavonol compounds such as kaempferol, quercitrin, isorhamnetin, isorhamnetin-3-O-glucoside, quercetin 3-galactoside, quercetin 3-O-glucuronide, and rutin, along with anthocyanins, exhibited markedly higher concentrations in red testa than in pink testa, potentially linked to the differential expression of *AhF3′H*.

The accumulation of metabolites hinges on the presence and activity of structural genes within metabolic pathways. In our investigation, we noted the absence of the CYP75A subfamily in peanuts, which includes flavonoid-3′,5′-hydroxylase (F3′5′H) [57]. While F3′H catalyzes dihydrokaempferol to produce dihydroquercetin and subsequently cyanidin, typically responsible for red or purple pigmentation. F3′5′H, also named as “the blue gene”, converts dihydrokaempferol into dihydromyricetin, leading to delphinidin production, a pigment associated with shades of blue [70]. This discrepancy likely elucidates why pink, red, and purple peanuts are commonplace, while blue peanuts remain absent.

## 5. Conclusions

In this study, a comprehensive analysis of the *CYP* gene superfamily in *A. hypogaea* was conducted, resulting in the identification of a total of 589 *AhCYPs*. Through phylogenetic analysis, chromosome localization, gene structure, promoter characterization, collinearity analysis, and gene expression pattern analysis, we revealed the distribution, classification, evolution, and expansion of AhCYP superfamily, as well as the specificity of its expression in testa. The qRT-PCR results involved in the flavonoid pathway of *AhCYPs* demonstrated that their expression had stage specificity during the development of peanut testa, and there were differences in pink and red testa. By combining the gene expression level with the amount of metabolite accumulation, we found that there was a direct correlation between them. This provides evidence to reveal the mechanism of differentiated accumulation of flavonoids in different color testa.

## Figures and Tables

**Table 1:** Statistics of CYP gene families in *A. hypogaea* and *A. thaliana*.

Clan Type	Clan Name	Family Name	Number of AhCYPs	Number of AtCYPs	Clan Type	Clan Name	Family Name	Number of AhCYPs	Number of AtCYPs
A-type	CYP71 clan	CYP701	4	1	non-A type	CYP72 clan	CYP709	0	3
		CYP703	2	1			CYP714	12	2
		CYP705	0	25			CYP715	7	1
		CYP706	4	7			CYP72	24	9
		CYP71	105	48			CYP721	0	1
		CYP712	4	2			CYP734	2	1
		CYP73	5	1			CYP735	4	2
		CYP75	6	1		CYP85 clan	CYP702	0	6
		CYP76	53	8			CYP707	11	4
		CYP77	3	4			CYP708	5	3
		CYP78	12	6			CYP716	14	2
		CYP79	20	8			CYP718	2	1
		CYP81	28	16			CYP720	3	1
		CYP82	50	5			CYP722	4	1
		CYP83	21	2			CYP724	2	2
		CYP84	26	1			CYP85	11	1
		CYP89	4	6			CYP87	4	1
		CYP93	27	1			CYP88	14	2
		CYP98	2	3			CYP90	11	3
non-A type	CYP51 clan	CYP51	3	1		CYP86 clan	CYP704	14	3
	CYP710 clan	CYP710	2	4			CYP86	6	10
	CYP711 clan	CYP711	4	1			CYP94	8	6
	CYP74 clan	CYP74	11	2			CYP96	29	13
	CYP97 clan	CYP97	6	3	Total			589	235

**Table 2:** AhCYP genes associated with flavonoid biosynthesis.

Num.	Gene ID	Subfamily Name	CYPs Name
1	*AhP3CRQW*	CYP93A	3,9-dihydroxypterocarpan 6a-hydroxylase (D6aH)
2	*AhQM5IC5*	CYP93A	
3	*AhK1LFJJ*	CYP73A	cinnamate 4-hydroxylase (C4H)
4	*AhSGZ2CH*	CYP73A	
5	*AhAYA1A5*	CYP73A	
6	*AhAM3FDB*	CYP75B or CYP706	flavonoid 3′-hydroxylase (F3′H)
7	*AhD5PZ9F*	CYP75B or CYP706	
8	*AhK8H9R8*	CYP75B	
9	*Ah8F7PE4*	CYP75B	
10	*Ah9J8RGD*	CYP81E	isoflavone 2′-hydroxylase (I2′H)
11	*Ah9P7KAZ*	CYP81E	
12	*AhXDA15Z*	CYP81E	
13	*Ah35X7CR*	CYP81E	isoflavone 3′-hydroxylase (I3′H)
14	*AhX1BDZQ*	CYP81E	
15	*AhZ630MX*	CYP81E	
16	*Ah1WY37S*	CYP93C	2-hydroxyisoflavanone synthase (IFS)
17	*AhB6E5H4*	CYP93C	
18	*AhQ2BYIT*	CYP93B	flavone synthase II (FNS II)
19	*AhQ2QWLN*	CYP93B	
20	*Ah57KEBM*	CYP71D	flavonoid 6-hydroxylase (F6H)
21	*AhD84CI6*	CYP71D	

## Data Availability

Not applicable.

## Supplementary Materials

The following supporting information can be downloaded at: https://www.mdpi.com/article/10.3390/genes14101944/s1, Figure S1: Seq Logos of 10 MEME-motifs for the identified AhCYP proteins; Figure S2. Gel graphics of total RNA of all samples; Figure S3. Melting curves of qRT-PCR in this study; Table S1: Sequences of the primers used in this study; Table S2: List of the 589 *AhCYP450* genes identified in this study; Table S3: Statistics on the number of *AhCYPs* on each peanut chromosome; Table S4: Length, size, and physicochemical properties of AhCYPs; Table S5: The information of conservative sequence in the *AhCYP* gene family; Table S6: *Cis*-element analysis of *AhCYP* gene promoters; Table S7: Gene structure information statistics of the *AhCYP* family; Table S8: Tandem duplicated *AhCYP* gene pairs; Table S9: Segmentally duplicated *AhCYP* gene pairs; Table S10: Syntenic relationships between peanut and other three species; Table S11: One-to-one orthologous relationships between *Arachis hypogaea* and other three species; Table S12: Transcriptome expression data of *AhCYPs* in four materials; Table S13: Determination of anthocyanin content; Table S14: The relative expression of *AhCYPs*.
