# Supplementary material for: Phylogenomic Analysis of Cytochrome P450 Gene Superfamily and Their Association with Flavonoids Biosynthesis in Peanut (Arachis hypogaea L.)
Source: Genes (Basel). 2023 Oct 15;14(10):1944. doi: 10.3390/genes14101944 (PMC10606413; doi:10.3390/genes14101944)
Supplement: Supplementary file 1 [file genes-14-01944-s001.zip › Figure S1.pptx]

## Slide 1
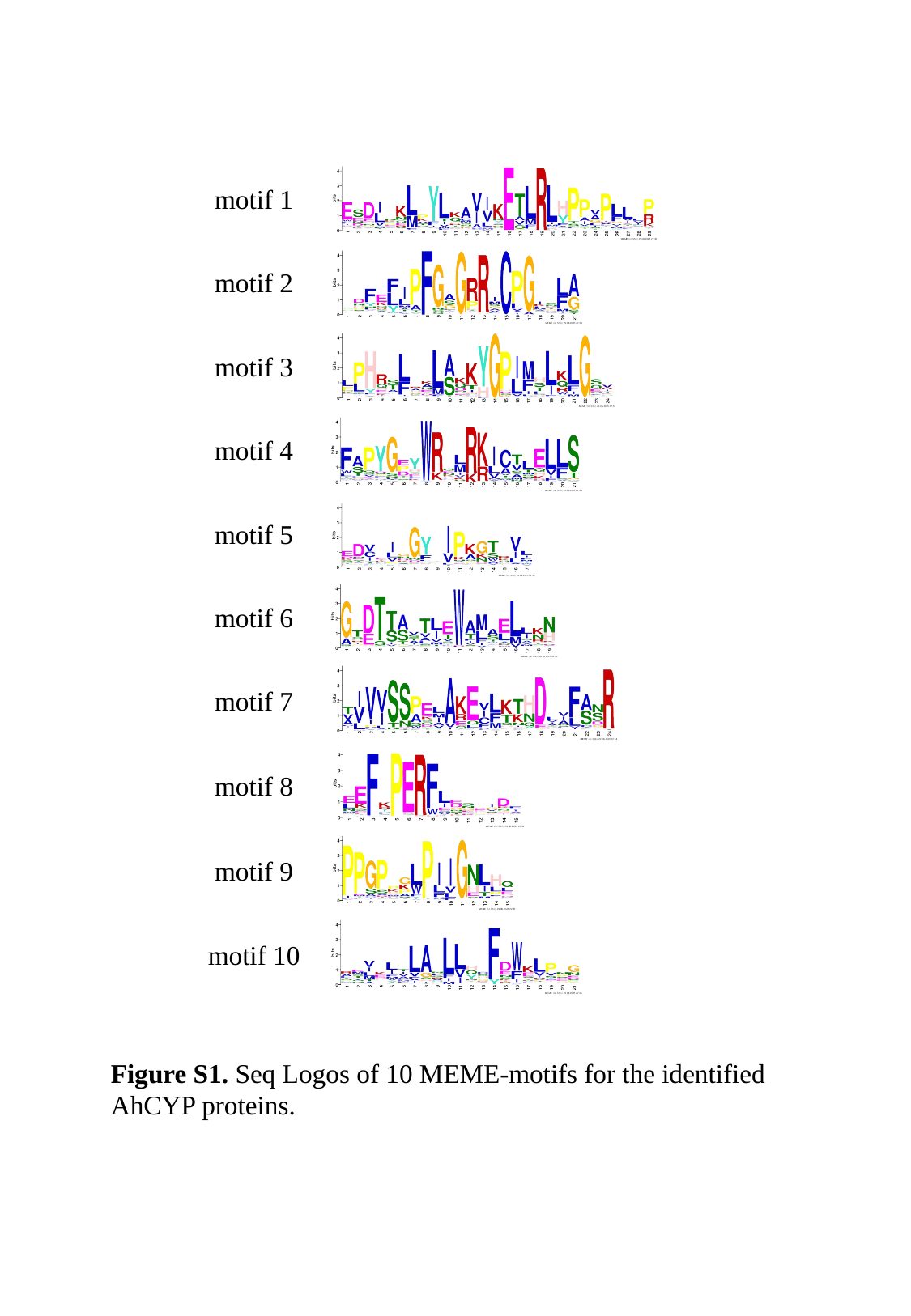

motif 1
motif 2
motif 3
motif 4
motif 5
motif 6
motif 7
motif 8
motif 9
motif 10
Figure S1. Seq Logos of 10 MEME-motifs for the identified AhCYP proteins.
