# Supplementary material for: Phylogenomic Analysis of Cytochrome P450 Gene Superfamily and Their Association with Flavonoids Biosynthesis in Peanut (Arachis hypogaea L.)
Source: Genes (Basel). 2023 Oct 15;14(10):1944. doi: 10.3390/genes14101944 (PMC10606413; doi:10.3390/genes14101944)
Supplement: Supplementary file 1 [file genes-14-01944-s001.zip › Figure S3.pptx]

## Slide 1
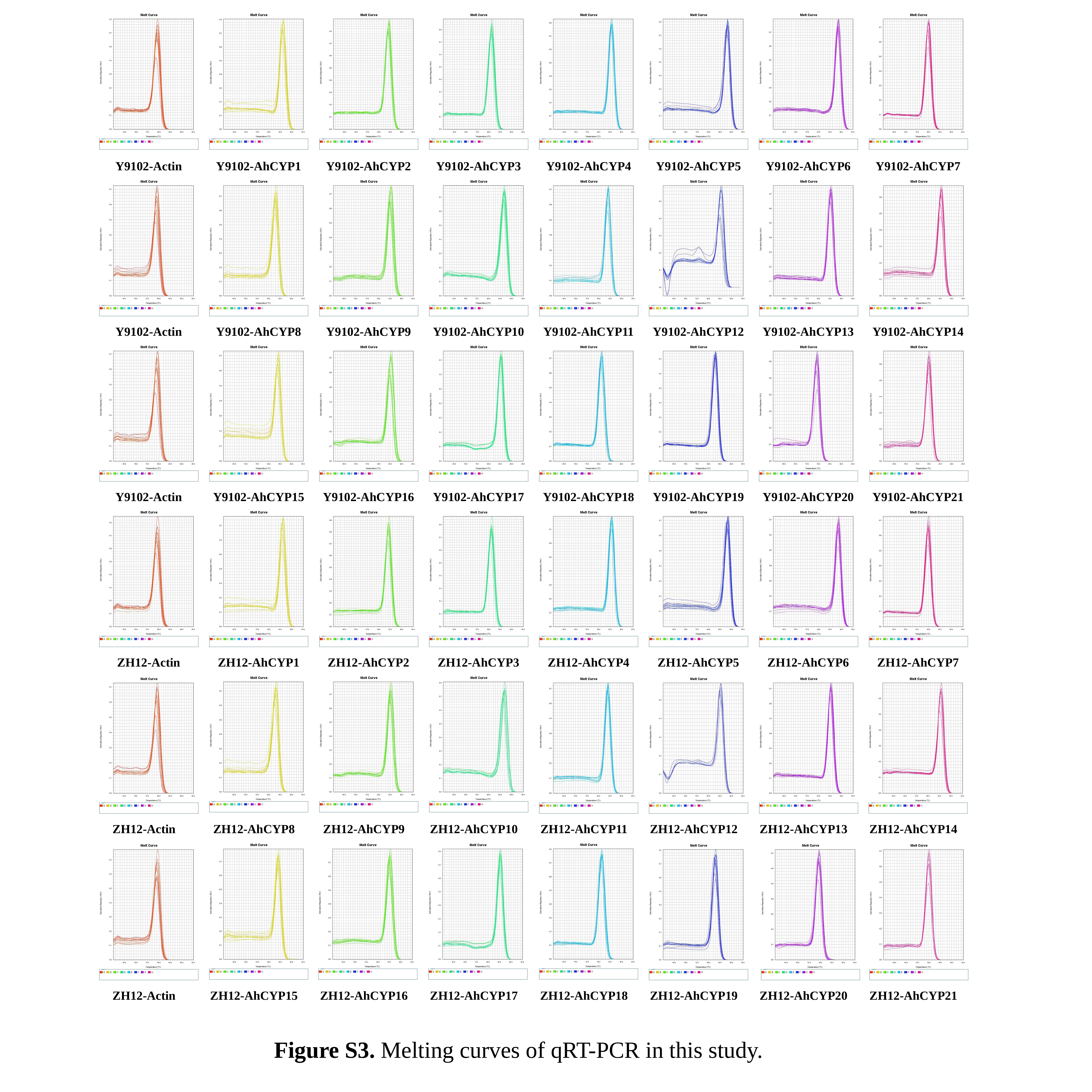

Y9102-AhCYP4
Y9102-AhCYP6
Y9102-AhCYP7
Y9102-AhCYP5
Y9102-AhCYP1
Y9102-AhCYP2
Y9102-AhCYP3
Y9102-Actin
Y9102-AhCYP11
Y9102-AhCYP13
Y9102-AhCYP14
Y9102-AhCYP12
Y9102-AhCYP8
Y9102-AhCYP9
Y9102-AhCYP10
Y9102-Actin
Y9102-AhCYP18
Y9102-AhCYP20
Y9102-AhCYP21
Y9102-AhCYP19
Y9102-AhCYP15
Y9102-AhCYP16
Y9102-AhCYP17
Y9102-Actin
ZH12-AhCYP4
ZH12-AhCYP6
ZH12-AhCYP7
ZH12-AhCYP5
ZH12-AhCYP1
ZH12-AhCYP2
ZH12-AhCYP3
ZH12-Actin
ZH12-AhCYP11
ZH12-AhCYP13
ZH12-AhCYP14
ZH12-AhCYP12
ZH12-AhCYP8
ZH12-AhCYP9
ZH12-AhCYP10
ZH12-Actin
ZH12-AhCYP18
ZH12-AhCYP20
ZH12-AhCYP21
ZH12-AhCYP19
ZH12-AhCYP15
ZH12-AhCYP16
ZH12-AhCYP17
ZH12-Actin
Figure S3. Melting curves of qRT-PCR in this study.
