# Supplementary figures and images for: Phylogenomic Analysis of Cytochrome P450 Gene Superfamily and Their Association with Flavonoids Biosynthesis in Peanut (Arachis hypogaea L.)
Source: Genes (Basel). 2023 Oct 15;14(10):1944. doi: 10.3390/genes14101944 (PMC10606413; doi:10.3390/genes14101944)

## Slide 1
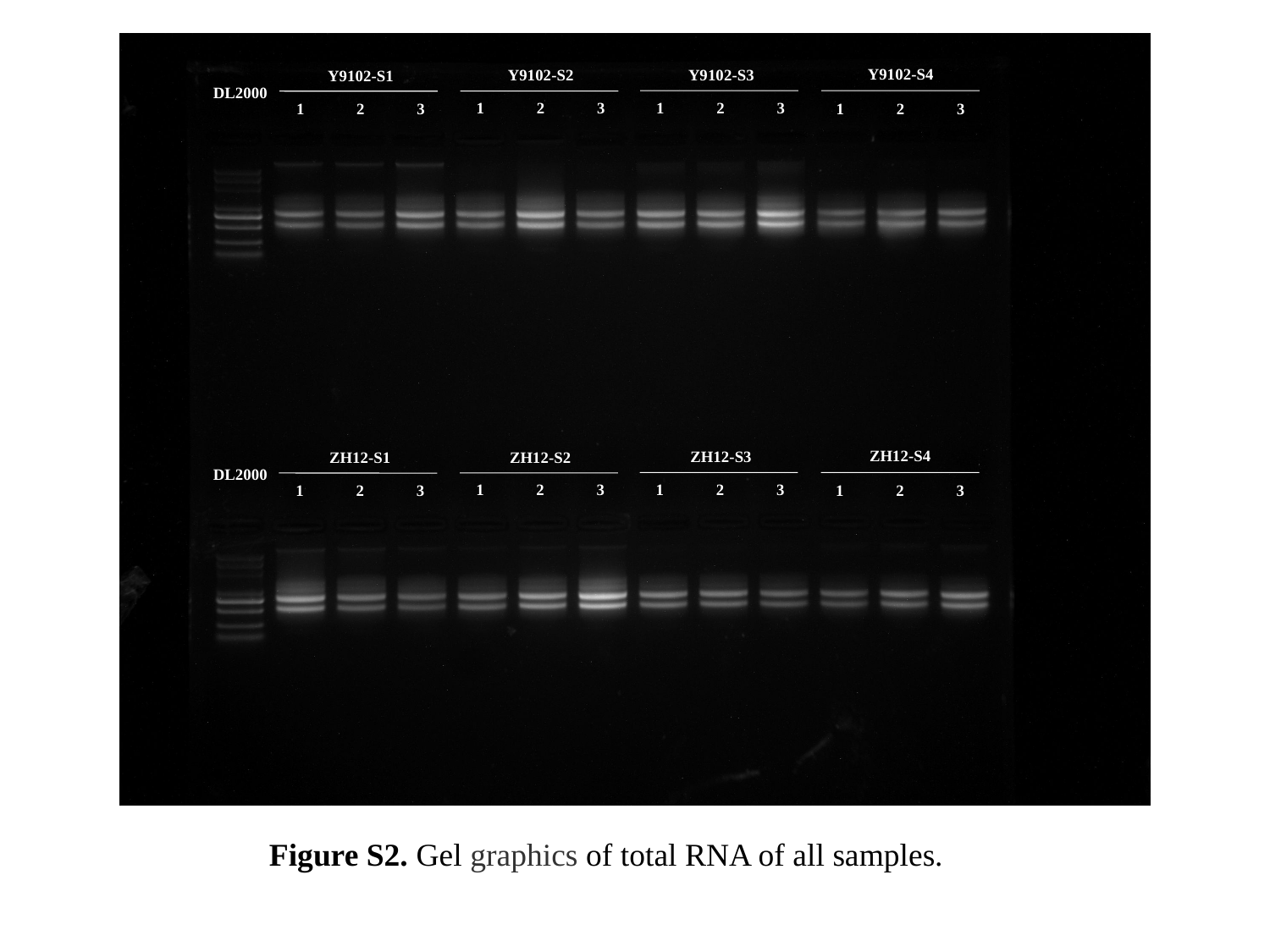

Y9102-S4
Y9102-S3
Y9102-S2
Y9102-S1
DL2000
1 2 3
1 2 3
1 2 3
1 2 3
ZH12-S4
ZH12-S3
ZH12-S2
ZH12-S1
DL2000
1 2 3
1 2 3
1 2 3
1 2 3
Figure S2. Gel graphics of total RNA of all samples.

Supplement: Supplementary file 1 [file genes-14-01944-s001.zip › Figure S2.pptx]
